# Upregulation of the TRPA1 Ion Channel in the Gastric Mucosa after Iodoacetamide-Induced Gastritis in Rats: A Potential New Therapeutic Target

**DOI:** 10.3390/ijms21165591

**Published:** 2020-08-05

**Authors:** Kata Csekő, Dániel Pécsi, Béla Kajtár, Ivett Hegedűs, Alexander Bollenbach, Dimitrios Tsikas, Imre László Szabó, Sándor Szabó, Zsuzsanna Helyes

**Affiliations:** 1 Department of Pharmacology and Pharmacotherapy, Medical School, University of Pécs, H-7624 Pécs, Hungary; zsuzsanna.helyes@aok.pte.hu; 2 Szentagothai Research Centre, University of Pécs, H-7624 Pécs, Hungary; 3 Institute for Translational Medicine, Medical School, University of Pécs, H-7624 Pécs, Hungary; daniel.pecsi@aok.pte.hu; 4 1st Department of Medicine, Medical School, University of Pécs, H-7624 Pécs, Hungary; szaboimi@yahoo.com; 5 Department of Pathology, Medical School, University of Pécs, H-7624 Pécs, Hungary; belakajtar@yahoo.com (B.K.); hegiv@citromail.hu (I.H.); 6 Hannover Medical School, Institute of Toxicology, Core Unit Proteomics, 30625 Hannover, Germany; bollenbach.alexander@mh-hannover.de (A.B.); tsikas.dimitros@mh-hannover.de (D.T.); 7 School of Medicine, University of California, Irvine, CA 92697, USA; sszabo@auhs.edu; 8 Department of Pharmaceutical Science, American University of Health Sciences, Signal Hill, CA 90755, USA; 9 PharmInVivo Ltd., H-7629 Pécs, Hungary

**Keywords:** TRPA1, TRV1, gastritis, inflammation, erosion, rodent

## Abstract

Acute gastritis is often untreatable by acid secretion-inhibiting drugs. Understanding the protective mechanisms including the role of Transient Receptor Potential Ankyrin1 (TRPA1) and Vanilloid1 (TRPV1) channels localized on capsaicin-sensitive afferents and non-neuronal structures might identify novel therapeutic approaches. Therefore, we characterized a translational gastritis model using iodoacetamide (IAA) and investigated TRPA1/V1 expressions. Wistar rats and CD1, C57Bl/6J mice were exposed to IAA-containing (0.05, 0.1, 0.2, 0.3, 0.5%) drinking water for 7 or 14 days. Body weight and water consumption were recorded daily. Macroscopic lesions were scored, qualitative histopathologic investigation was performed, TRPA1/V1 immunopositivity and mRNA expressions were measured. IAA induced a concentration-dependent weight loss and reduced water intake in both species. Hyperemia, submucosal edema, inflammatory infiltration and hemorrhagic erosions developed after 7 days, while ulcers after 14 days in rats. *Trpa1* mRNA/protein expressions were upregulated at both timepoints. Meanwhile, TRPV1 immunopositivity was upregulated in the gastric corpus after 0.05% IAA ingestion, but downregulated after 0.2%, whereas *Trpv1* mRNA did not change. Interestingly, no macroscopic/microscopic changes were observed in mice. These are the first data for the concentration- and duration-dependent changes in the IAA-induced gastritis in rats accompanied by TRPA1 upregulation, therefore, its therapeutic potential in gastritis should further be investigated.

## 1. Introduction

Gastric mucosal injury can be exhibited by various forms of macroscopic and histopathological alterations, such as diffuse hyperemia, inflammation, erosion, or even hemorrhagic ulcerations. Several attempts have been made to classify the different types of gastritis, but it is difficult due to the complexity of its pathophysiological mechanisms [1]. There is often no correlation between the symptoms and the macroscopic lesions or histopathological changes [2]. Based on its localization, the injury can be diffuse, antrum- or corpus-predominant, or even multifocal. Regarding the duration, we can differentiate between acute or chronic forms [3]. However, the etiology of the condition is at least as important.

Several drugs may alleviate the gastric lesions induced by chemical factors such as the non-steroidal anti-inflammatory drugs (NSAID), alcohol or bile reflux, *Helicobacter pylori* infection or irradiation [4]. The gold standard treatment is often limited to acid secretion inhibitors, such as proton pump inhibitors or histamine H2 receptor antagonists, since enhancing cytoprotective mechanisms is challenging [5]. The gastroprotective effect of capsaicin-sensitive peptidergic sensory neurons innervating the gastric mucosa has long been investigated by our group [6,7,8].

Recently special focus has been directed towards the Transient Receptor Potential Vanilloid 1 (TRPV1, “capsaicin receptor”) and Ankyrin 1 (TRPA1) ion channels located and often co-expressed mainly on these sensory fibers, which are identified as novel anti-inflammatory, analgesic and gastroprotective targets. These polymodal nociceptors play an important role in thermo-, mechanical- and chemo-sensation, as well as neurogenic inflammation and hyperalgesia [9,10,11,12]. Besides the exogenous agents, especially spices, like capsaicin (the pungent agent of chili pepper), cinnamaldehyde, allyl isothiocyanate (mustard oil) and allicin, they are activated by several endogenous agents as lipoxygenase products, bradykinin, and protons produced in the inflammatory tissues [10,12,13]. Their activation leads to the release of pro-inflammatory neuropeptides, such as calcitonin gene-related peptide (CGRP) and substance P (SP) resulting in vasodilation, plasma protein extravasation and inflammatory cell activation (neurogenic inflammation) [14,15]. Meanwhile, anti-inflammatory, analgesic and cytoprotective mediators—most importantly somatostatin—are also released from the same nerve ending, which inhibit inflammation and tissue damage both locally and systemically [8,16].

TRPV1 is also present on several non-neuronal structures in the gastrointestinal tract, such as the gastrin-secreting parietal and gastric epithelial cells, as well as the esophageal, small intestinal and colonic epithelial cells [17,18]. TRPA1 is less extensively studied, but its expression was described in isolated crypts and epithelial cells of the colon, as well as small intestinal neuroendocrine cells [18,19,20]. Moreover, both receptors were reported on CD4^+^ T cells emphasizing their role in sensory-immune interactions [21,22].

The role of TRPV1 in gastrointestinal mucosal defense mechanisms is virtually controversial. Studies with *Trpv1* and *Trpa1* gene-deficient mice show contradictory data about their roles in colitis, most likely depending on the key pathomechanisms of the different colitis models [23]. *Trpa1* and *Trpv1* gene deletion decreased dextran sulfate sodium (DSS)-induced colitis severity [24], as well as *Il10^−/−^*-induced spontaneous colitis in *Trpv1*-deficient mice [21], whereas *Trpv1*-deficient mice exhibited more severe colitis in the dinitrobenzene sulfonic acid (DNBS)-induced model [25]. The protective role of TRPA1 has also been described in the *Il10^−/−^*-induced spontaneous colitis model [22]. Low dose of the TRPV1 activator capsaicin is protective against the alcohol- and indomethacin-induced gastric mucosal injury [26], it reduces basal gastric acid secretion and enhances gastric emptying [27]. In contrast to TRPV1, little is known about the expression changes and role of TRPA1 in the stomach [28].

Animal models are important for the molecular investigation of gastric injury, since these models may reveal very early biochemical and molecular alterations, much before microscopic or macroscopic lesions can be seen. Good models should have translational relevance. However, in virtually all animal models of gastric injury (e.g., NSAID-, stress-induced) the lesions are well circumscribed (i.e., superficial erosions and/or deep ulcers). Gastritis in humans, on the other hand, is a diffuse inflammatory damage involving all or most parts of the stomach [1]. Iodoacetamide (IAA) is a water soluble sulfhydryl alkylating chemical, which, by depleting sulfhydryl groups, including the protective antioxidant glutathione (GSH) in the gastric mucosa, allows reactive oxygen species production and oxidative tissue damage [29]. The reduced GSH plays an essential role in maintaining mucosal integrity [30]. Nitric oxide synthase (NOS) also contributes to mucosal protection via the production of nitric oxide (NO), which increases mucosal blood flow like other gastroprotective compounds [31,32]. IAA may interfere with NOS activity, thus also affecting gastric mucosal integrity. Reactive oxygen species react with various cell components including cell membrane, mitochondria and DNA, potentially leading to cell death/necrosis, which triggers neutrophil recruitment [33,34].

Therefore, our aim was to characterize a translationally relevant gastritis model using the irreversible sulfhydryl-group blocker IAA and to investigate the expression changes of TRPA1 and TRPV1 in this model.

## 2. Results

### 2.1. Macroscopic Evaluation of Rat Gastric Mucosa

Gastritis was induced by the administration of 0.05%, 0.1% or 0.2% IAA solution in the drinking water of Wistar rats, littermates drinking IAA-free tap water served as control animals. Rats were euthanized at days 7 and 14, their stomachs were harvested and opened along the greater curvature.

**Figure 1:**
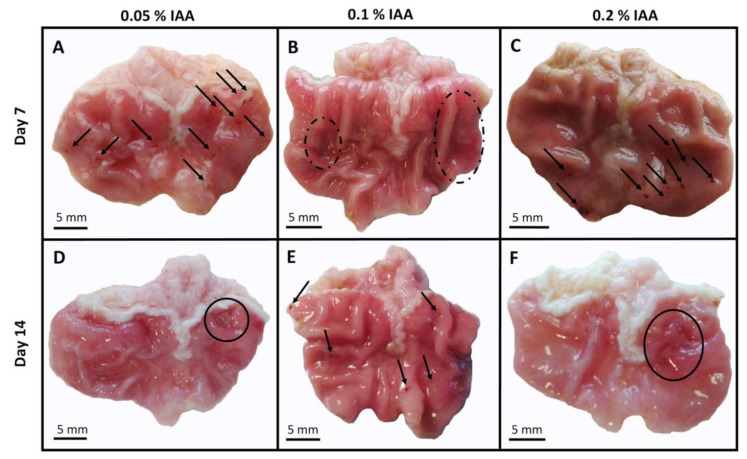
Macroscopic pictures of iodoacetamide-induced (IAA) gastric mucosal inflammation. Representative photos of gastric mucosa of Wistar rats receiving (**A**) 0.05%, (**B**) 0.1%, (**C**) 0.2% IAA for 7 and (**D**–**F**) for 14 days, respectively. After 7 days, diffuse hyperemia (dashed circles) and several superficial mucosal erosions (black arrows) developed, while by the end of the 14-day-long protocol, chronic ulcers were also observable in some animals (circles on panel (**D**,**F**)).

**Figure 2:**
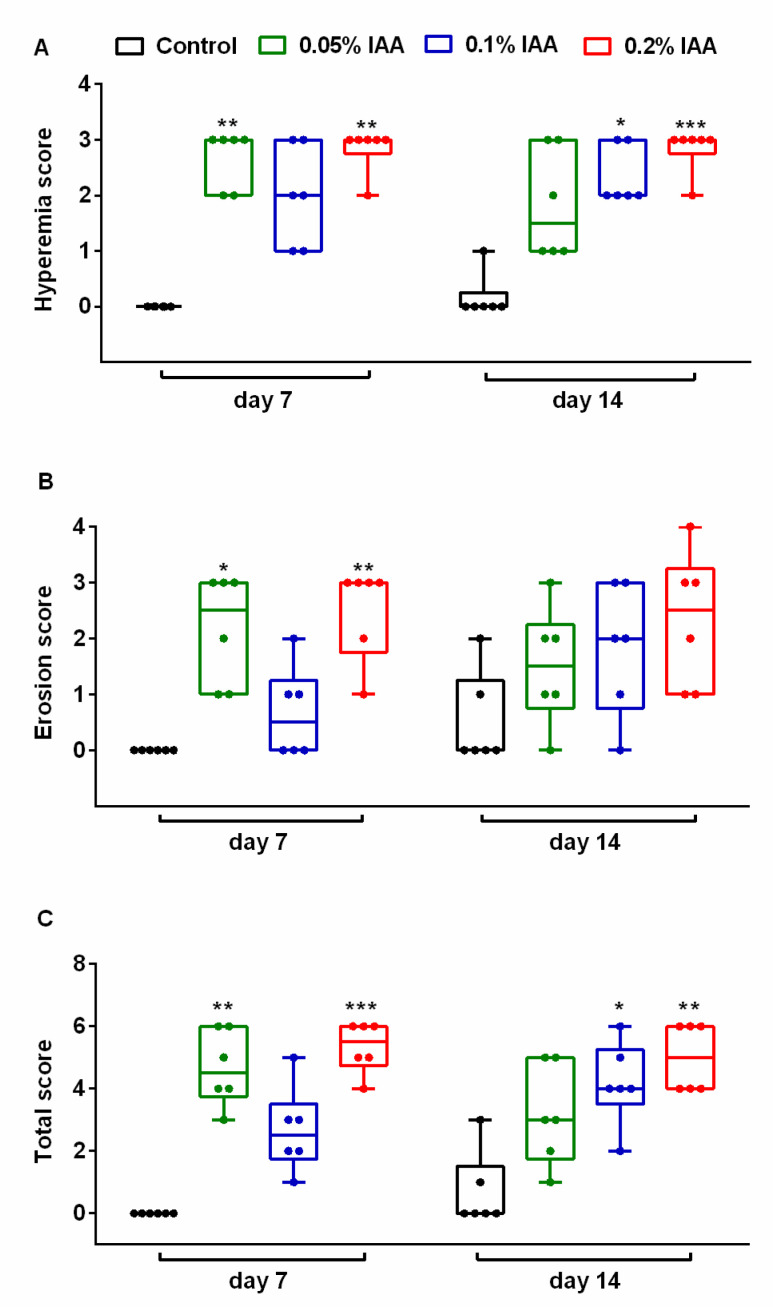
Semi-quantitative macroscopic evaluation of gastric mucosa in Wistar rats. Macroscopic findings, such as (**A**) hyperemia, (**B**) superficial erosions/ulcers and (**C**) total score were evaluated semi-quantitatively. 0.05% IAA induced significant lesions after 7 days, which were less pronounced after 14 days, whereas in the 0.1% IAA-treated group, the peak in macroscopic changes was observed after 14 days. 0.2% IAA induced hyperemia, and erosions developed at both timepoints, although there was no significant difference in macroscopic picture by IAA concentrations. Box plots represent minimum, first quartile, median, third quartile, and maximum values with individual data plots; *n* = 6/group. (Kruskal–Wallis followed by Dunn’s multiple comparison test to observe intergroup differences at given timepoints, * *p* < 0.05, ** *p* < 0.005, *** *p* < 0.001 vs. control group; Mann–Whitney test was performed to analyze intragroup differences by time—not significant).

Extensive hyperemia, mucosal hemorrhage and several erosions or superficial ulcers were observed at both timepoints in all three examined concentrations. Semi-quantitative analysis showed significant hyperemic areas and erosions in both 0.05% and 0.2% IAA-treated groups at day 7 compared to the controls. At day 14, lesions, especially the extent of the hyperemic area in the 0.1% and 0.2% IAA-treated groups were significantly greater. Macroscopic changes showed no significant difference either by the increasing concentrations of ingested IAA or by time; however, ulcerations were more pronounced after 14 days of IAA-drinking (Figure 1 and Figure 2).

### 2.2. Microscopic Alterations in the Inflamed Rat Gastric Mucosa

**Figure 3:**
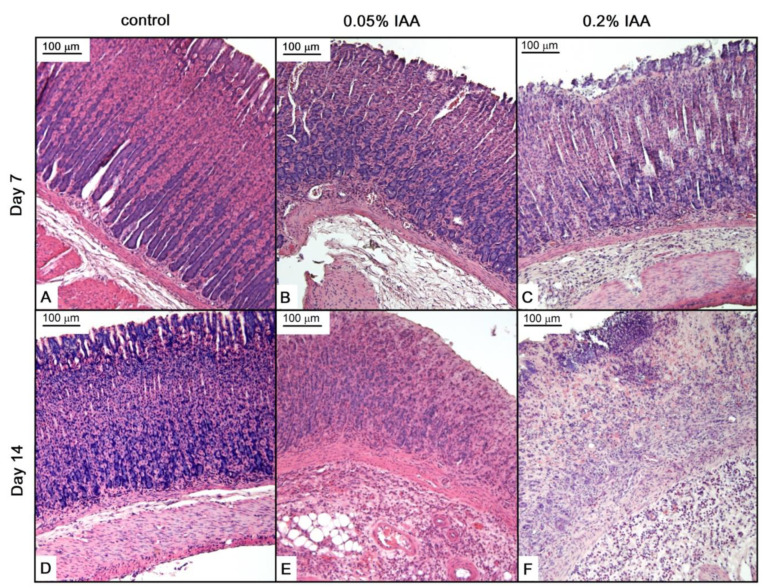
IAA treatment induced microscopic alterations in Wistar rat gastric mucosa. Representative HE-stained microscopic pictures of (**A**), (**D**) control; (**B**,**E**) 0.05% IAA-treated, and (**C**,**F**) 0.2% IAA-treated rat gastric mucosa at days 7 and 14, respectively.

Seven days of IAA treatment resulted in submucosal widening due to massive edema. In higher concentrations, extensive inflammatory cell infiltration was also observed. After 14 days, focal epithelial cell sloughing/erosions, and in some areas, almost total mucosal necrosis involving the muscularis mucosae were seen, admixed with acute and chronic inflammatory cells, both in the mucosa and the submucosa (Figure 3).

### 2.3. Weight Change and Water Consumption

**Figure 4:**
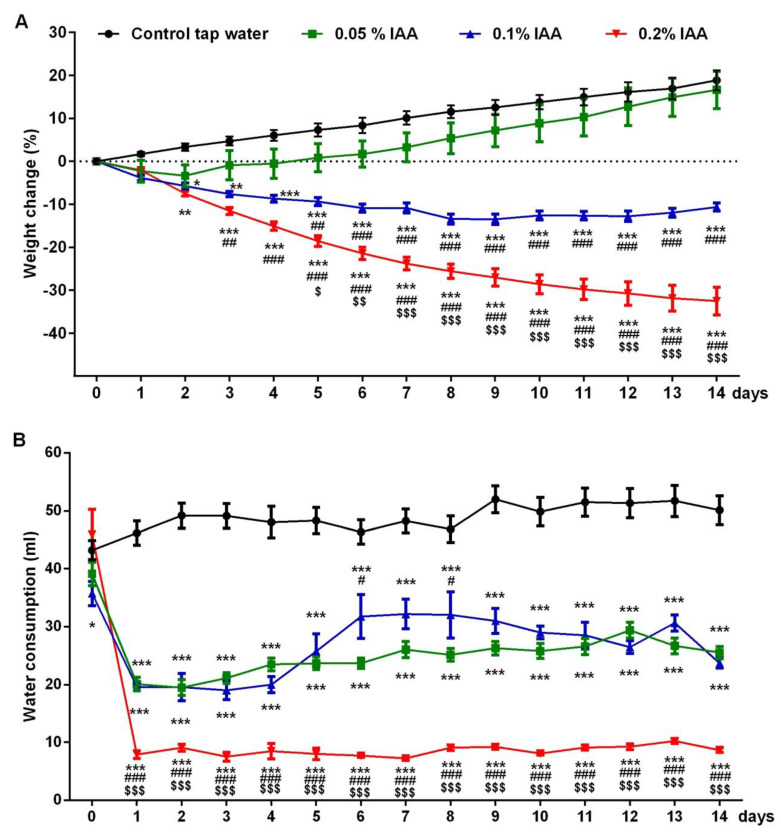
IAA-induced body weight change and water intake in Wistar rats. (**A**) IAA administration resulted in a dose-dependent weight loss and (**B**) reduced water intake in Wistar rats. Data are shown as means ± SEM; *n* = 6/group (repeated measures two-way ANOVA followed by Bonferroni’s modified t-test; * *p* < 0.05, ** *p* < 0.005, *** *p* < 0.001 vs. control group; # *p* < 0.05 ## *p* < 0.005 ### *p* < 0.001 vs. 0.05% IAA group; $ *p* < 0.05 $$ *p* < 0.05 $$$ *p* < 0.001 vs. 0.1% IAA group).

In Wistar rats, IAA induced a concentration-dependent weight change. Similarly to vehicle-treated animals, low concentration (0.05%) of IAA resulted in ~15% weight gain by the end of the 14-day experiment. Meanwhile, 0.1% and 0.2% IAA induced a concentration-dependent, gradual weight loss with a maximum of 13.4 ± 1.2% and 32.5 ± 3.3%, respectively (Figure 4A). The total water consumption of the 0.05% and the 0.1% IAA-treated groups was halved compared to the control group, and it was even more decreased to around 9 mL daily in the case of 0.2% concentration (Figure 4B).

### 2.4. GSH Concentration of the Rat Stomach Tissue

The GSH content of the rat gastric mucosa was measured to be (mean ± standard deviation) 3.64 ± 1.91 nmol/mg protein in the control group, 5.82 ± 3.94 nmol/mg protein in the group receiving 0.1% IAA for 7 days, and 5.71 ± 2.07 nmol/mg protein in the group receiving 0.1% IAA for 14 days (data not shown). There were no statistically significant differences between the groups (*p* = 0.343, one-way ANOVA). The content of free GSH measured by us has also been reported by other groups for gastric mucosa in rats [35].

**Figure 5:**
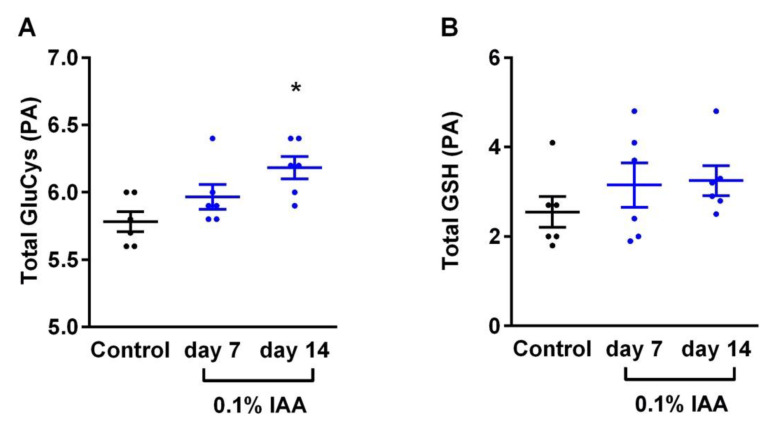
(**A**) Total glutathione (GSH) and (**B**) Total GSH (tGSH) γ-glutamyl-cysteine levels of the rat gastric mucosa measured by HPLC analysis. Data are shown as mean ± SEM; *n* = 6/group (ordinary one-way ANOVA; * *p* < 0.05).

*N*-acetylcysteine ethyl ester (NACET) is a useful reagent to reduce disulfides to their sulfhydryl compounds. Previously, we have shown that NACET is useful for the measurement of tGSH [36]. IAA treatment increased tGSH (based on the peak area in mAU of the GSH peak) numerically, but not significantly in the rat gastric mucosa: 2.55 ± 0.85 in the control group; 3.15 ± 1.21 in the group treated with 0.1% IAA for 7 days; and 3.25 ± 0.82 in the group treated with 0.1% IAA for 14 days (Figure 5B). tGluCys increased significantly (between one week and two weeks: 5.78 ± 0.18; 5.97 ± 0.23; 6.18 ± 0.20, respectively) (Figure 5A). These changes resulted in a decrease in the tGluCys/tGSH molar ratio, which was not significant: 2.43 ± 0.72; 2.13 ± 0.77; 1.98 ± 0.39, respectively (data not shown).

### 2.5. Quantification of TRPA1 and TRPV1 Immunopositivity

**Figure 6:**
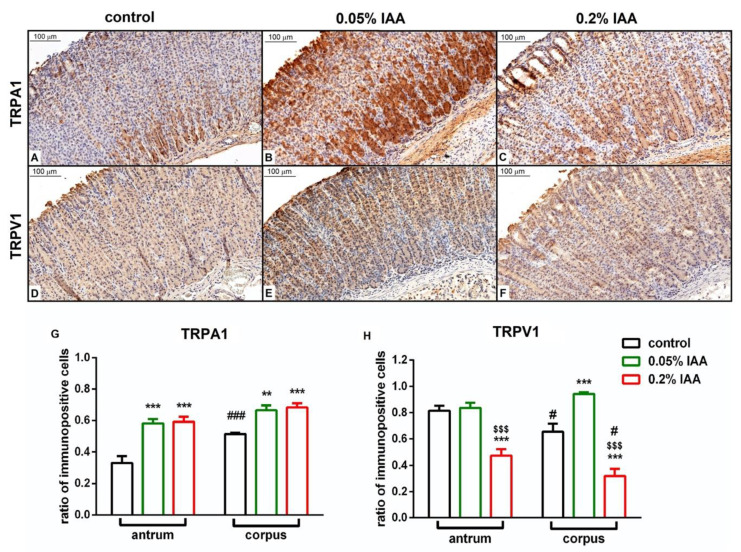
Quantitative analysis of TRPA1 and TRPV1 immunohistochemistry. Representative pictures of (**A**–**C**) TRPA1 (antrum) and (**D**–**F**) TRPV1 (corpus) immunohistochemistry of rat gastric mucosa (**A**,**D**) under control conditions, and 14 days after (**B**,**E**) 0.05% IAA, and (**C**,**F**) 0.2% IAA treatment. Panel (**G**,**H**) demonstrates the quantitative histopathological analysis of TRPA1 and TRPV1 immunohistochemistry, respectively, calculated by the ratio of immunopositive cells/total cell number. Data are shown as means ± SEM; *n* = 6 animals/group, 10 fields of vision/slide/animal; ordinary two-way ANOVA followed by Dunn’s multiple comparisons test ** *p* < 0.005, *** *p* < 0.001 vs. control group; $$$ *p* < 0.001 vs. 0.05% IAA-treated group; # *p* < 0.05, ### *p* < 0.001 vs. respective antrum samples.

Mild TRPA1 and strong TRPV1 immunopositivity was detected on the epithelial cells in the intact control samples. Quantification, as shown by the ratio of immunopositive cells, revealed a significant upregulation of TRPA1 after both 0.05% and 0.2% IAA administration by day 14 in both the antrum and corpus epithelial cells. Although TRPV1 immunopositivity also increased in the corpus, but did not change in the antrum in the case of 0.05% IAA, it significantly decreased in both localizations after 0.2% IAA treatment (Figure 6).

### 2.6. Trpa1, Trpv1 Relative Gene Expression Changes in the Inflamed Rat Stomach

**Figure 7:**
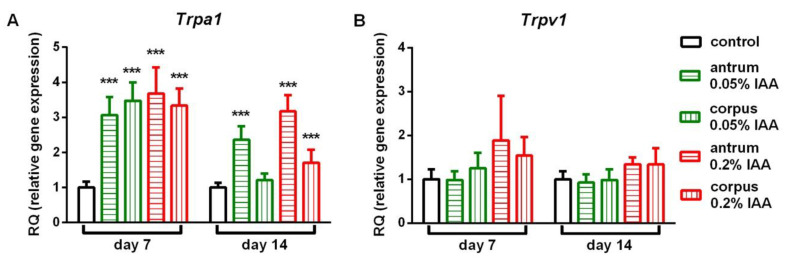
Relative gene expression of *Trpa1* and *Trpv1.* mRNA levels of (**A**) *Trpa1* were significantly upregulated after 0.05% and 0.2% IAA treatment, whereas (**B**) *Trpv1* gene expression did not show significant alterations either by time, concentration or localization. Data are shown as means ± SEM; *n* = 6/group; Student’s unpaired *t*-probe *** *p* < 0.001 vs. control group.

In agreement with the TRPA1 protein expression, *Trpa1* mRNA was significantly upregulated in both 0.05% and 0.2% IAA-treated groups after 7 and 14 days as well, however, there was no detectable alteration in *Trpv1* relative gene expression either by time, dose or localization (Figure 7).

### 2.7. IAA-Induced Alterations in Mice

CD1 and C57Bl/6J mice were administered 0.1%, 0.3%, or 0.5% IAA solution in the drinking water for 7 or 14 days; littermates drinking IAA-free tap water served as control animals.

**Figure 8:**
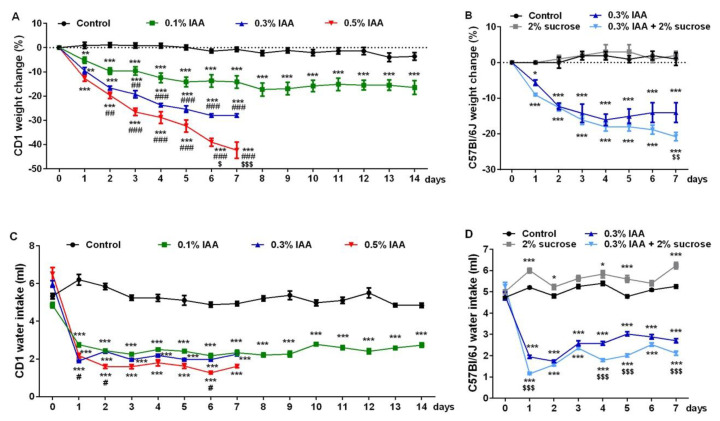
IAA-induced weight change and water intake in mice. IAA administration resulted in (**A**,**B**) a dose-dependent weight loss and (**C**,**D**) significantly reduced water intake in both CD1 and C57Bl/6J mice, respectively. Data are shown as means ± SEM; *n* = 6/group (repeated-measures ANOVA followed by Bonferroni’s modified *t*-test; * *p* < 0.05, ** *p* < 0.005, *** *p* < 0.0005 vs. control group; # *p* < 0.05, ## *p* < 0.005, ### *p* < 0.0005 vs. 0.1% IAA group, $ *p* < 0.05, $$ *p* < 0.005, $$$ *p* < 0.0005 vs. 0.3% IAA group).

In CD1 mice, we observed an IAA dose-dependent continuous, gradual weight loss; in the 0.3% and 0.5% IAA-treated groups, weight reduction was so severe at the end of the 7-day-long protocol, that a 14-day-long protocol could not be performed due to the ethical considerations of humane endpoints (Figure 8A,C). Although water intake was significantly reduced in all IAA-treated groups, it showed no concentration-dependence, and could not explain the remarkable dose-dependent weight loss of these animals.

C57Bl/6J mice proved to be more resistant to 0.3% IAA, which induced ~14% weight loss after 7 days, half as much as the same concentration in CD1 mice (~28%). Interestingly, adding 2% sucrose to 0.3% IAA significantly reduced both the fluid intake, as well as the weight (~21%) of C57Bl/6J mice compared to the 0.3% IAA-drinking group (Figure 8B,D).

Surprisingly, in contrast to Wistar rats, where we observed similar body weight change and decreased water intake, no macroscopic lesions or microscopic alterations were present in either of the mouse groups drinking IAA (Table 1).

## 3. Discussion

This is the first comprehensive and comparative acute and chronic diffuse gastritis model study, in which IAA-induced concentration- and duration-dependent changes were described in Wistar rats. IAA induced concentration-dependent weight loss and gastric erosions already after 7-day ingestion of IAA in drinking water accompanied by massive submucosal edema and extensive infiltration by acute and chronic inflammatory cells, and subsequently, hemorrhagic erosions. After 14 days, ulcers were observed as deep necrosis involving the muscularis mucosae, which was more severe and more extensive in rats with high (0.2%) IAA concentration in their drinking water.

IAA is a sulfhydryl alkylating agent, which inhibits free radical scavenging by depleting reduced GSH, thus inducing gastric injury [29]. However, although this mechanism of action is the state of the art, GSH concentrations are rarely measured directly in the stomach mucosa. In our present study, we did not find GSH reduction in response to IAA application. This might be due to the fact that we measured it after 7 or 14 days of IAA ingestion when the lesions were already fully developed or started to heal. Furthermore, we used the whole stomach tissue, not only the mucosa. Nevertheless, the small, but significant increase in tGluCys also supports the onset of the healing phase with the elevation of oxidative stress and/or GSH synthesis enzyme activity. IAA-induced rapid GSH depletion was demonstrated in cultured Wistar rat astrocytes [37], rat hepatocytes [38,39], and in human erythrocytes as well [39]. GSH content from in vivo experiments are highly influenced by the complex inflammatory/oxidative/antioxidant regulatory system of the animal, as well as the differences in sampling protocols. In an IAA-induced gastritis model, 0.1% IAA induced a robust, almost 4-fold increase in gastric mucosal GSH after one week, whereas in the same experimental paradigm, an approximately 50% decrease was measured after two weeks of IAA administration [40]. The robust GSH increase was accompanied by a similarly elevated MPO activity, which is a reliable indicator of inflammatory cell infiltration/activation, suggesting that at the time of lesion formation, the fine regulation is also activated to counteract the imbalance of the aggressive/defensive factors. Similar rebound GSH increase was reported on alveolar epithelial cells [41], where genes involved in GSH synthesis, such as γ-glutamylcysteine transpeptidase, and activator protein-1 (AP-1) were upregulated as an adaptive mechanism.

Only a few studies have used the IAA gastritis model, but with different paradigms: 1) various durations (5 days–25 weeks) [42,43,44,45], 2) rats weighing 100–500 g [43,46], 3) different strains [42,44,46], 4) different IAA concentrations [33,44,45,47], 5) routes of administration, even with additional sucrose in drinking water [33,34,44,47]. Therefore, the comparison of the outcomes and conclusions of these different studies is not easy, but they are consistent in myeloperoxidase (MPO) elevation and macroscopic/microscopic alterations characteristic to diffuse gastritis with hemorrhages. We did not observe significant changes in the severity of lesions regarding hyperemia and erosions between 7 and 14 administration days in agreement with the literature [34], although ulcer formation was more pronounced after 14 days.

In this well-characterized gastric erosion/ulcer inflammatory model, our major finding is that both 0.05% and 0.2% IAA ingestion induced *Trpa1*, but not *Trpv1* mRNA upregulation in the rat antrum and corpus after 7 days—that remained elevated by the end of the 14-day period.

Most activators of the TRPA1 channel are structurally diverse molecules, which suggests that their effect is not exerted based on the conventional lock-and-key principle. They act as reactive electrophile compounds (allylisothiocyanate, cinnamaldehyde) inducing covalent reversible modifications of the cytoplasmic N terminal of the receptor [48,49]. IAA being a cysteine-modifying alkylating compound is able to bind covalently to the reactive cytoplasmic cysteine residues, thus inducing TRPA1 activation as demonstrated in HEK cell culture by Ca^2+^ imaging [48]. However, TRPA1 upregulation in our experiments is not explained simply by direct IAA-evoked receptor activation or desensitization, since prolonged administration of IAA to human TRPA1-expressing cells was described not to induce receptor desensitization [50]. Moreover, IAA acts as a partial TRPA1 agonist, since after its continuous administration, a subsequently applied other agonists (*para*-benzoquinone) that induce rapid desensitization by itself further increased TRPA1-mediated Ca^2+^ current [50]. Therefore, IAA-induced TRPA1 expression increase in the stomach is more likely due to the inflammatory cascade, which is further supported by its upregulation in water immersion restraint, stress-induced acute gastric mucosal ulcerations in rats [51].

There are no data on the expression and function of the TRPA1 channel in gastritis and only little information is available on TRPV1. The distribution of TRPV1 immunopositivity was reported to be increased in chronic gastritis biopsies, however, in that study, control samples were collected from patients with functional dyspepsia [52]. Their investigation is more thorough in inflammatory bowel disease, their expression changes and the role are virtually controversial, but the overall function is likely to be protective [23].

TRPV1/A1 expression on capsaicin-sensitive peptidergic nerve endings and non-neural cells, such as gastric epithelial and inflammatory cells [18,19,21,22], also shown by our present results detecting TRPA1 mRNA in the stomach, make the interpretation of their roles much more complex. Several endogenous inflammatory mediators activating TRPV1 (protons, lipoxygenase products) are produced during the IAA-induced inflammatory reaction, which might also influence TRPA1 function and expression since their interactions have been described [22,53,54].

The role of capsaicin-sensitive sensory nerves in IAA-induced gastritis has been investigated by defunctionalizing these neurons with high doses of capsaicin [47]. The role of these peptidergic afferents depends on the experimental paradigm and the consequent pathophysiological mechanisms—they can both inhibit (e.g., ethanol-induced gastritis) or aggravate the inflammation presumably via SP and CGRP release (e.g., IAA-induced gastritis), underlining the role of neurogenic component in inflammation [47]. The role of TRPA1 and TRPV1 in gastritis might also be attributed to the mediation of inflammatory visceral hyperalgesia and abdominal pain. IAA was shown to significantly increase Na^+^ current in the dorsal root ganglia of T9 and T10 afferent neurons [55], and enhanced visceromotor responses primarily by increased activity of the splanchnic nerves [46].

Surprisingly, mice (both CD1 and C57Bl/6J strains) proved to be resistant to all applied concentrations of IAA, even higher than the most damaging one in the rat. Although they also exhibited concentration-dependent weight loss similar to the rat, no macroscopic or microscopic changes have been found in the stomach. The few studies coming from one group point out the lack of IAA-induced macroscopic lesions in mice supporting our present results, but describe a mixed inflammatory infiltration, characteristic to mild gastritis [56]. Interestingly, most of these studies showed that after an initial weight loss, mice recovered by the third day of administration, although their water intake was reduced by approximately 50% throughout the study [57,58,59]. This is also in agreement with our observation, that body weight loss cannot be explained solely by less drinking in IAA-treated animals. The concentration-dependent reduction in fluid consumption suggests an oral aversion that might be due to the potential gastro-irritating effect of the colorless, odorless IAA solution.

Since TRPA1 is activated by IAA, it raises the question whether the known species differences in sequence homology, as well as its selectivity to a range of ligands, might contribute to the observed species differences in the IAA model. As discussed above, IAA contains a highly reactive electrophilic moiety lacking structural selectivity, that forms alklylation adducts by binding to the cysteine residues on the N-terminal of TRPA1 [49], which might potentially lead to its activation [48]. Therefore, it is more likely, that species differences in the IAA-induced diffuse gastritis model is not due to the heterogeneity in TRPA1 ion channel sequence, and that TRPA1 upregulation is rather a consequence of tissue injury. Determining resistance mechanisms was beyond the scope of our study; however, it might provide valuable information on gastroprotective mechanisms yet not fully known. As a general limitation of all immunohistochemical techniques, TRPA1- and TRPV1-like immunopositivity determined on the histopathological sections in our study might not provide direct evidence for the receptor protein expression. However, (i) the parallel receptor mRNA changes, (ii) the positive control with dorsal root ganglia samples, (iii) the lack of immunopositivity with the blocking peptides provided by the producers, (iv) as well as the extensive use of both antibodies in the literature [60,61] and in our earlier studies [18,62,63] suggest the reliability and validity of our IHC results supporting our conclusion.

Here, we show the first results on the upregulation of the TRPA1 ion channel in a well-characterized translational gastric injury model in correlation with duration-dependent macroscopic and microscopic lesions. These data will provide a good basis for evaluating the effect of TRPA1-targeting pharmacological interventions on the different components of the gastric injury.

## 4. Materials and Methods

### 4.1. Animals and Ethics

Experiments were performed on 8-week-old male CD1 and C57Bl/6J mice weighing 18–25 g and Wistar rats weighing 180–220 g at the beginning of the study; each group consisted of 6 animals. Animals were bred and kept in the Laboratory Animal House of the Department of Pharmacology and Pharmacotherapy, University of Pécs, at 24–25 °C, provided with standard rodent chow and water ad libitum, maintained under 12 h light-dark cycle. All procedures were carried out according to the 40/2013 (II.14.) Government Regulation on Animal Protection and Consideration Decree of Scientific Procedures of Animal Experiments and Directive 2010/63/EU of the European Parliament. They were approved by the Ethics Committee on Animal Research of University of Pécs according to the Ethical Codex of Animal Experiments (license no.: BA02/2000-20/2019, 27 June 2019).

### 4.2. Experimental Protocol

Gastritis was induced by the administration of IAA (Sigma-Aldrich Inc., Darmstadt, Germany) to the drinking water. Since IAA is light-sensitive, IAA-containing drinking water was prepared freshly every day by dissolving 0.1, 0.2, 0.4, 0.6 or 1 g IAA in 200 mL tap water (0.05%, 0.1%, 0.2%, 0.3% and 0.5% concentration, respectively) for 7 or 14 days consecutively, depending on the experimental paradigm.

### 4.3. Study in Rats

**Figure 9:**
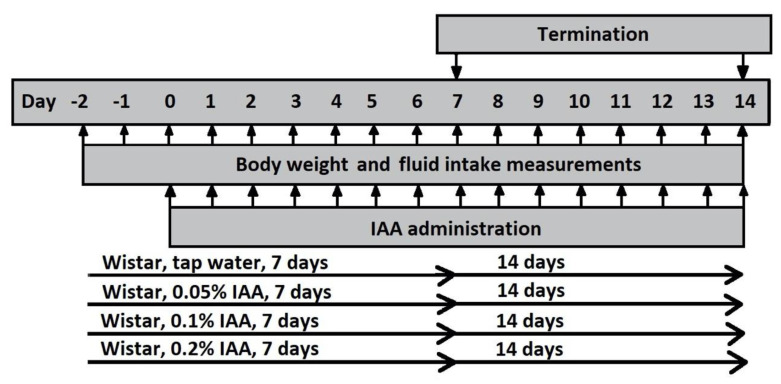
Experimental design of IAA gastritis with Wistar rats. Male Wistar rats were divided into 8 groups according to the duration of the protocol (7 days or 14 days) and concentration (0.05%, 0.1% or 0.2%) of IAA dissolved in drinking water. Control rats received tap water. Body weight and fluid intake were monitored daily throughout the experiment. At the end of the protocol, rats were euthanized and their stomachs were excised for macroscopic, microscopic evaluation and further molecular biological assessment.

Rats were randomized into 8 groups of 6 animals in each, and received 0.05%, 0.1% or 0.2% IAA solution in the drinking water for 7 or 14 days consecutively. Littermates drinking IAA-free tap water served as control animals (Figure 9).

### 4.4. Study in Mice

**Figure 10:**
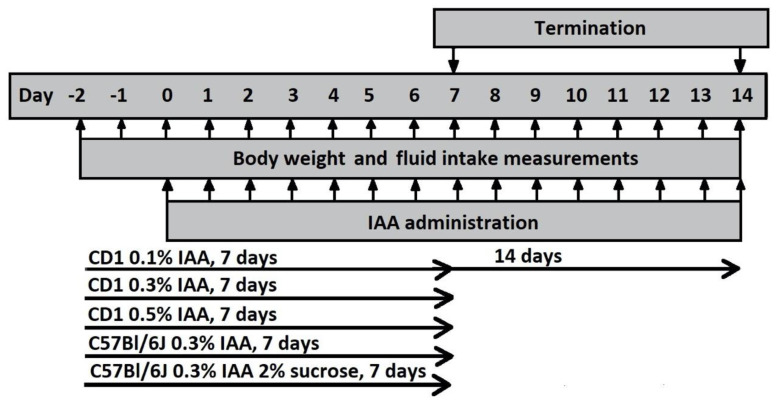
Experimental design of IAA gastritis with CD1 and C57Bl/6J mice. Male CD1 and C57Bl/6J mice were divided into groups receiving various concentrations (0.1%, 0.3% and 0.5%) of IAA dissolved in drinking water. Control mice received tap water. Body weight and fluid intake were monitored daily throughout the experiment. At the end of the protocol, mice were euthanized and their stomachs were excised for macroscopic and microscopic evaluation.

In the first mouse study, CD1 mice were randomized into 4 groups: mice receiving 0.1% IAA for 7 and 14 days, with the respective control groups. Based on the negative results of this study, CD1 mice were randomized in 3 groups receiving 0.3% and 0.5% IAA for 7 days; the control group drank tap water. To investigate interstrain differences, C57Bl/6J mice were randomized into 4 groups receiving (1) 0.3% IAA-containing drinking water, (2) 0.3% IAA-containing drinking water, which also contained 2% sucrose, (3) a control group drinking tap water, and (4) a second control group receiving 2% sucrose dissolved in tap water (Figure 10).

Fluid intake and body weight were measured daily in each study. At the end of the study, animals were euthanized under ketamine (120 mg/kg ip.; Calypsol, Gedeon Richter Plc., Budapest, Hungary) and xylazine (6 mg/kg ip.; Sedaxylan, Eurovet Animal Health B.V., Bladel, The Netherlands) anesthesia. The stomach was excised, opened along the greater curvature and rinsed with room temperature saline. After photo documentation of the macroscopic lesions, the stomach was cut in four sections: antrum and corpus specimens were fixed in 6% formaline and 5 μm sections were stained with hematoxylin and eosin (H&E) for histopathologic and immunohistochemical evaluation. Other antral and corpus samples were snap-frozen for molecular biologic assessments.

### 4.5. Macroscopic and Microscopic Evaluations of IAA-Induced Gastric Lesions

The extent and severity of the macroscopic lesions were evaluated by a semiquantitative scoring system based on the extent of hyperemia (0: none; 1: <25%, 2: 25–50%; 3: >50% of the total gastric mucosa) and the number of erosions/ulcerations (0: none; 1: 1–2; 2: 3–4; 3: ≥5). Excised gastric samples were paraformaldehyde-fixed (4%) and embedded in paraffin, 5 μm sections were cut and stained with H&E for further qualitative histological analysis, i.e., the assessment of the extent of lesions, submucosal infiltration and capillarization.

### 4.6. GSH Measurement

Total GSH (tGSH) and total γ-glutamyl-cysteine (tGluCys) were measured in gastric mucosa specimens by a specific HPLC method with RP 18 NUCLEOSHELL HPLC columns (Macherey-Nagel, Düren, Germany) after incubation with *N*-acetylcysteine ethyl ester (for detailed methodological description see (Supplementary Material (Figure S1)).

### 4.7. TRPV1 and TRPA1 Immunohistochemistry and Scoring

For antigen recovery, the paraformaldehyde-fixed and paraffin-embedded tissue samples were deparaffinized, rehydrated and incubated in acidic citrate buffer (pH 6) in a microwave oven. Endogenous peroxidase activity was quenched 3% hydrogen peroxide. The sections were washed and incubated in blocking solution, then treated with a 1:1000 dilution of rabbit polyclonal anti-TRPA1 (ab68848; Abcam, Cambridge, UK) and anti-TRPV1 (GP14100; Neuromics, Edina, MN, USA) antibodies. Slides were incubated with anti-rabbit secondary antibody conjugated with HRP (DakoCytomation, Carpinteria, CA, USA) with the EnVision system. The reaction was visualized by 0.01% hydrogen peroxide containing 3,3-diaminobenzidine tetrachloride, and histological counterstaining was performed with hematoxylin [64]. Quantitative assessment of TRPA1 and TRPV1 immunopositivity was performed based on the % ratio of the immunopositive cells on 10 fields of vision/slide/animal by an expert pathologist blinded to the study. Pannoramic Digital Slide Scanner with CaseViewer software (3DHISTECH Ltd., Budapest, Hungary) was used for both the evaluation and taking the representative photos of the slides. Incubating untreated rat gastric mucosa with Tris-buffered saline instead of the primary antibodies served as the negative control, while sections of rat dorsal root ganglia expressing TRPA1 and TRPV1 abundantly were used as positive controls. Antibody selectivity was validated by the lack of immunopositivity after the respective blocking peptides (ab150297 for TRPA1; Abcam, Cambridge, UK and P14100 for TRPV1; Neuromics, Edina, MN, USA) and was also based on literature data [65].

### 4.8. Determination of Trpv1 and Trpa1 Relative Gene Expression

Total RNA was purified by TRI Reagent (Molecular Research Center Inc., Cincinnati, OH, USA) with Direct-Zol RNA MiniPrep isolation kit (Zymo Research, Irvine, CA, USA) following the manufacturer’s protocol. The quantity and purity of RNA samples were assessed by NanoDrop ND-1000 spectrophotometer (NanoDrop Technologies Inc., Wilmington, DE, USA) and then treated with deoxyribonuclease I enzyme (Zymo Research, Irvine, CA, USA). Purified total RNA (100 ng) was reverse transcribed with Maxima First Strand cDNA Synthesis Kit (Thermo Fisher Scientific, Waltham, MA, USA) according to the manufacturer’s instructions. Real-time qPCR was conducted on a Stratagene Mx3000P qPCRSystem (Agilent Technologies, Santa Clara, CA, USA) using Luminaris HiGreen LowROX qPCR Master Mix (Thermo Fisher Scientific, Waltham, MA, USA) to amplify transcripts. The following primer pairs were used: the reference gene glyceraldehyde 3-phosphate dehydrogenase (*Gapdh*) (NM_017008.4) (sense): 5′-TGCACCACCAACTGCTTAGC-3′ and (antisense): 5′-GGCATGGACTGTGGTCATGAG-3′; *Trpv1* (NM_031982.1) (sense): 5′-AATACACCATCGCTCTGCT-3′ and (antisense): 5′-CAATGTGCAGTGCTGTCTGG-3′; *Trpa1* (NM_207608.1) (sense): 5′-ATCCAAATAGACCCAGGCACG-3′ and (antisense): 5′-CAAGCATGTGTCAATGTTTGGTACT-3′. Primers with similar efficiencies were used. In order to verify primer specificity, dissociation curve analyses were performed. All reactions were measured in triplicates, and the geometric mean of their Ct values were calculated. The determination of relative messenger RNA (mRNA) expression levels was performed according to the comparative DDCt method using samples of control animals as calibrator.

### 4.9. Statistical Analysis

Statistical analysis was performed by using GraphPad Prism v6 software. Values for all measurements are expressed as means ± SEM of *n* = 6 animals in each group. Evaluation of body weight change and fluid intake was performed by repeated-measures ANOVA followed by Bonferroni’s modified *t*-test. Semiquantitative macroscopic scoring was analyzed by the non-parametric Kruskal–Wallis method followed by Dunn’s multiple comparison test to observe intergroup differences at given timepoints, while Mann–Whitney test was performed to analyze intragroup differences by time. GSH measurements were analyzed by one-way ANOVA, while TRPA1 and TRPV1 immunopositivities were evaluated by ordinary two-way ANOVA followed by Dunn’s multiple comparisons test. qPCR measurements were evaluated by Student’s unpaired *t*-test.

## Figures and Tables

**Table 1:** Summary of IAA administration-induced alterations in mice.

Strain	IAA Conc.	Duration	Weight Loss	Water Intake	Macroscopic and Microscopic Picture
CD1	0.1%	14 days	~17%	↓	negative
CD1	0.1%	7 days	~14%	↓	negative
CD1	0.3%	7 days	~28%	↓	negative
CD1	0.5%	7 days	~42%	↓↓	negative
C57Bl/6J	0.3%	7 days	~14%	↓	negative
C57Bl/6J	0.3% + 2% sucrose	7 days	~21%	↓↓	negative

## Supplementary Materials

Supplementary materials can be found at https://www.mdpi.com/1422-0067/21/16/5591/s1. Methods. GSH measurements. GSH results measured by GC-MS. Figure S1. Peak area ratio (PAR) of m/z 269/272 for pyroglutamate (pGlu) (A), of m/z 301/307 for glutamate (Glu) (B) and Glu-to-pGlu ratio (C) measured by GC-MS in intact and 0.1% IAA-treated stomach samples. Data are shown as mean ± SEM; *n* = 6/group (ordinary one-way ANOVA; * *p* < 0.05) [36,66,67,68].
