# Supplementary material for: Upregulation of the TRPA1 Ion Channel in the Gastric Mucosa after Iodoacetamide-Induced Gastritis in Rats: A Potential New Therapeutic Target
Source: Int J Mol Sci. 2020 Aug 5;21(16):5591. doi: 10.3390/ijms21165591 (PMC7460663; doi:10.3390/ijms21165591)
Supplement: Supplementary file 1 [file ijms-21-05591-s001.pdf]

International Journal of
Molecular Sciences

MDPI

Supplementary Material

Methods

GSH Measurements

Glutathione (GSH) was measured in gastric mucosa specimens by a specific HPLC method after pre-column derivatization with *ortho*-phthaldehyde (OPA) as described previously for erythrocytic GSH [36]. The procedure for gastric mucosa GSH is described below in detail. With the exception of the HPLC analyses, which were all performed at room temperature on an Agilent 1100 Series system equipped with a UV detection and an autosampler Gerstel MPS-3, all other experimental procedures, including homogenization and ultrafiltration, were performed under cooling in ice bath to protect GSH from oxidation.

Wet specimens weighing 122 to 241 mg were homogenized in 1 mL aliquots of ice-cooled phosphate buffered saline (PBS, 0.1 M, pH 7.2) in precellys tubes (Bertin Instruments, Rockville, MD, USA) four times each for 20 s at 5500 rpm. The homogenates were immediately centrifuged (5 min, 3345× *g*, 4 °C), and the supernatants (500 µL) were ultrafiltered by centrifugation using Vivaspin 500 cartridges (15,000× *g*, 20 min, 4 °C; Sartorius, Goettingen, Germany). The ultrafiltrate samples (25–40 µL aliquots) were frozen immediately and kept stored at −20 °C until subsequent analysis. After thawing, 20 µL aliquots of the ultrafiltrates were diluted with 180 µL of ice-cooled PBS. Aliquots (50 µL) of the dilutions were treated with 900 µL borate buffer (0.4 M, pH 8.5) and 50 µL of the OPA reagent [36]. HPLC analysis with UV absorbance detection (338 nm) was performed 2 min after derivatization by injecting 20 µL aliquots of the derivatized samples.

The 5 µm RP 18 NUCLEOSHELL HPLC columns (guard column, 4 × 3 mm; analytical column, 250 × 4 mm) were from Macherey-Nagel (Düren, Germany) and were thermostated at 20 °C. Isocratic elution was performed with 10 vol% methanol-90 vol% sodium acetate (150 mM, pH 7.5) at a flow rate of 1 mL/min [36]. The GSH-OPA derivative eluted from the column at 3.924 ± 0.027 min (coefficient of variation, 0.7%). The concentration of GSH in the samples was calculated by using a calibration curve generated in the range 0–1000 µM upon sample dilution as performed for the ultrafiltrate samples. Plotting of the peak area of the GSH-OPA peak (y, mAU/min) versus the GSH concentration (x, µg/mL) resulted in a line ($r^2 = 0.9987$) with the regression equation $y = 1.38 + 0.31x$. The calibration curve samples were re-analyzed next day by HPLC and resulted in the regression equation $y = 0.53 + 0.33x$ ($r^2 = 0.9997$), indicating the high inter-assay precision of the method. This HPLC method can also analyze γ-glutamyl-cysteine (GluCys), but not cysteine and other cysteine-containing species [36]. In some samples, we observed a peak eluting in front of GSH-OPA peak (retention time, 2.964 ± 0.156 min) and is likely to be the GluCys-OPA derivative [66,67]. HPLC analysis of the GSH calibration curve samples did not reveal presence of GluCys in the GSH preparation. Incubation of biological samples including human erythrocytes with *N*-acetylcysteine ethyl ester (NACET) allows for the measurement of total GSH (tGSH) [36] and total GluCys (tGluCys) [66]. tGluCys includes free GluCys and GluCys released by NACET from symmetric and asymmetric disulfides (R-GluCys). Analogous, tGSH includes free GSH and GSH released by NACET from the symmetric GSH disulfide (i.e., GSSG) and from asymmetric disulfides (R-GSH). The GSH concentration in the homogenate samples was corrected by the protein concentration in the homogenates as measured by the BCA method and is reported as nmol GSH per mg wet protein. Due to the lack of a GluCys reference compound, the concentration of GluCys in the samples was not measured. Yet, it is assumed that GSH and GluCys, at a molar basis, yield closely similar peak areas.

We confirmed the GSH levels obtained by HPLC analysis also with the help of gas chromatography mass spectrometry (GC-MS). We analyzed GSH in the gastric mucosa of untreated and treated rats after its in situ conversion to pyroglutamate (pGlu, 5-oxo-proline), as described earlier [68]. 10 µL aliquots of ultrafiltrates (cut off, 10 kDa) of rat gastric mucosa homogenate samples prepared in ice-cold 67 mM phosphate buffered saline (pH 7.4) were used. The samples were first

derivatized with 2 M HCl/CH3OH (60 min, 80 °C), then spiked with the internal standard hexadeutero-GSH and then acylated by pentafluoropropionic anhydride (30 min, 65 °C).

GSH Results Measured by GC-MS

There were no differences between the three rat groups with respect to pGlu, Glu/Glu-Cys or their molar ratio (see Supplementary Figure S1).

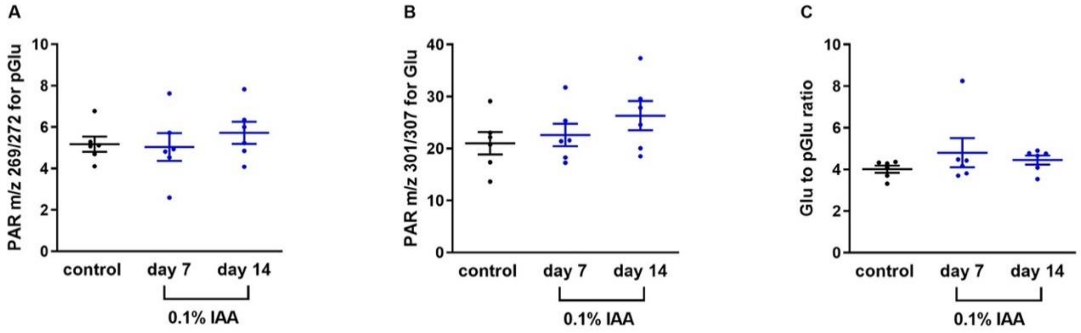

Figure S1. Peak area ratio (PAR) of m/z 269/272 for pyroglutamate (pGlu) (**A**), of m/z 301/307 for glutamate (Glu) (**B**) and Glu-to-pGlu ratio (**C**) measured by GC-MS in intact and 0.1% IAA-treated stomach samples. Data are shown as mean ± SEM; $n = 6$/group (ordinary one-way ANOVA; * $p < 0.05$).
